# Wrist Band Photoplethysmography Autocorrelation Analysis Enables Detection of Atrial Fibrillation Without Pulse Detection

**DOI:** 10.3389/fphys.2021.654555

**Published:** 2021-05-07

**Authors:** Eemu-Samuli Väliaho, Pekka Kuoppa, Jukka A. Lipponen, Juha E. K. Hartikainen, Helena Jäntti, Tuomas T. Rissanen, Indrek Kolk, Hanna Pohjantähti-Maaroos, Maaret Castrén, Jari Halonen, Mika P. Tarvainen, Onni E. Santala, Tero J. Martikainen

**Affiliations:** ^1^School of Medicine, Faculty of Health Sciences, University of Eastern Finland, Kuopio, Finland; ^2^Doctoral School, Faculty of Health Sciences, University of Eastern Finland, Kuopio, Finland; ^3^Department of Applied Physics, University of Eastern Finland, Kuopio, Finland; ^4^Heart Center, Kuopio University Hospital, Kuopio, Finland; ^5^Center for Prehospital Emergency Care, Kuopio University Hospital, Kuopio, Finland; ^6^Heart Center, North Karelia Central Hospital, Joensuu, Finland; ^7^Emergency Medicine, Faculty of Medicine, University of Helsinki, Helsinki, Finland; ^8^Department of Emergency Medicine and Services, Helsinki University Hospital, Helsinki, Finland; ^9^Department of Clinical Physiology and Nuclear Medicine, Kuopio University Hospital, Kuopio, Finland; ^10^Department of Emergency Care, Kuopio University Hospital, Kuopio, Finland

**Keywords:** atrial fibrillation, atrial fibrillation detection, arrhythmia detection, pulse detection, photoplethysmography, autocorrelation, algorithms, stroke

## Abstract

Atrial fibrillation is often asymptomatic and intermittent making its detection challenging. A photoplethysmography (PPG) provides a promising option for atrial fibrillation detection. However, the shapes of pulse waves vary in atrial fibrillation decreasing pulse and atrial fibrillation detection accuracy. This study evaluated ten robust photoplethysmography features for detection of atrial fibrillation. The study was a national multi-center clinical study in Finland and the data were combined from two broader research projects (NCT03721601, URL: https://clinicaltrials.gov/ct2/show/NCT03721601 and NCT03753139, URL: https://clinicaltrials.gov/ct2/show/NCT03753139). A photoplethysmography signal was recorded with a wrist band. Five pulse interval variability, four amplitude features and a novel autocorrelation-based morphology feature were calculated and evaluated independently as predictors of atrial fibrillation. A multivariate predictor model including only the most significant features was established. The models were 10-fold cross-validated. 359 patients were included in the study (atrial fibrillation *n* = 169, sinus rhythm *n* = 190). The autocorrelation univariate predictor model detected atrial fibrillation with the highest area under receiver operating characteristic curve (AUC) value of 0.982 (sensitivity 95.1%, specificity 93.7%). Autocorrelation was also the most significant individual feature (*p* < 0.00001) in the multivariate predictor model, detecting atrial fibrillation with AUC of 0.993 (sensitivity 96.4%, specificity 96.3%). Our results demonstrated that the autocorrelation independently detects atrial fibrillation reliably without the need of pulse detection. Combining pulse wave morphology-based features such as autocorrelation with information from pulse-interval variability it is possible to detect atrial fibrillation with high accuracy with a commercial wrist band. Photoplethysmography wrist bands accompanied with atrial fibrillation detection algorithms utilizing autocorrelation could provide a computationally very effective and reliable wearable monitoring method in screening of atrial fibrillation.

## Introduction

Atrial fibrillation (AF) is the most common tachyarrhythmia and it’s prevalence is increasing as the population ages (Morillo et al., 2017). AF is associated with thromboembolic complications, such as stroke (Xiong et al., 2015; Morillo et al., 2017; Pereira et al., 2020). It is estimated that 20–30% of all strokes are due to AF (Kirchhof et al., 2016; Pereira et al., 2020). In addition, 25% of ischaemic strokes are of unknown cause and there is persuasive evidence that most of these are of thromboembolic origin (Hart et al., 2014). Up to two thirds of strokes can be prevented with anticoagulation (Saxena and Koudstaal, 2004; Hart et al., 2007). A clinical challenge is that AF is often asymptomatic or paroxysmal (Xiong et al., 2015) and therefore, difficult to be diagnosed. Intermittent electrocardiograms (ECGs) recorded during clinical visits have a low likelihood of detecting paroxysmal AF. Long-term, continuous monitoring with automatic AF detection would improve AF screening detection allowing appropriate primary and secondary strategies for prevention of stroke (Pereira et al., 2020).

Photoplethysmography (PPG) technology is widely used for welfare or sport-tracking purposes. PPG has also been proven to be promising also in the detection of AF (Tison et al., 2018; Dörr et al., 2019; Fan et al., 2019; Kashiwa et al., 2019; Väliaho et al., 2019; Pereira et al., 2020). Usually, in PPG the rhythm assessment is based on pulse-to-pulse interval detection. However, AF detection with PPG based on pulse-to-pulse (PP) interval irregularity is often challenging. Namely, AF is characterized by poorly coordinated atrial activation, resulting in highly irregular heart rate and variable pulse wave amplitudes. In addition, the signal is susceptible to artifacts caused by motion of the sensor against the skin or poor sensor contact (Pereira et al., 2020). Furthermore, the pulse detection accuracy is lower in patients with AF compared to those in sinus rhythm (SR) and even lower in patients with episodes of short duration of AF (Väliaho et al., 2019). Several companies are currently developing wrist worn PPG devices with arrythmia detection features. Thus, reliable methods for PPG-based AF detection are under strong interest and could lead to improved rhythm diagnostics of AF patients.

In this study we introduce a novel PPG pulse wave morphology-based method which enables AF detection without the need of individual pulse detection. A robust morphology-based PPG-analysis can significantly improve AF detection accuracy of PPG wrist bands.

## Materials and Methods

### Study Design

The study was a national multi-center clinical study implemented in three hospitals in Finland: Kuopio University Hospital (KUH), Helsinki University Hospital (HUS) and North Karelia Central Hospital (NKCH). The data were combined from two studies (Afib study and Single-ECG study), both of which were approved by the Ethics Committee of KUH (237/2017 and 850/2018) and registered in the ClinicalTrials.gov database (NCT03721601^1^ and NCT03753139^2^).

The participants were given written and oral information and an opportunity to ask questions about the study. All participants provided written informed consent.

### Study Population

**FIGURE 1:**
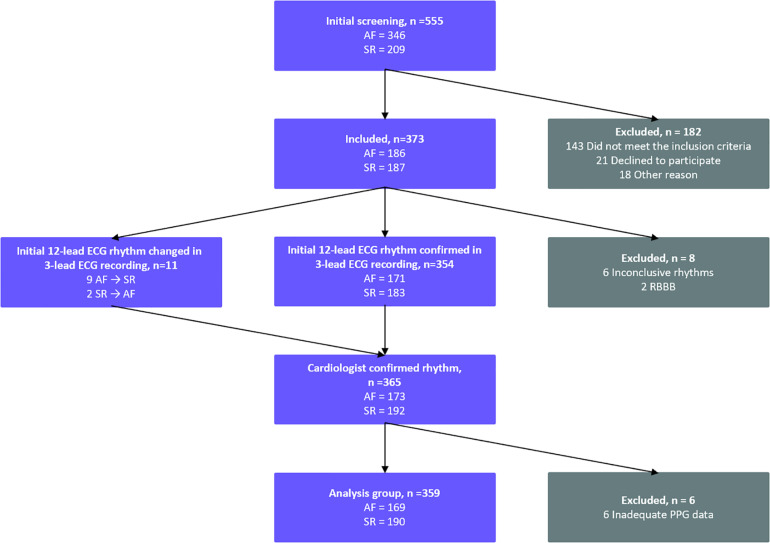
Standards for Reporting Diagnostic Accuracy Studies (STARD) flow diagram of the study patient flow. A total of 555 patients were screened in the participating hospitals KUH, HUS, and NKCH. 359 patients were included in the analysis. AF, atrial fibrillation; ECG, electrocardiogram; SR, sinus rhythm; PPG, photoplethysmography; RBBB, right bundle branch block.

A total of 555 patients were screened in the emergency care departments and the cardiological wards of the three participating hospitals (Figure 1) in two broader research projects (the Afib study and the Single-ECG study). A total of 295 patients were screened in KUH, HUS and NKCH between May – September 2017 (the Afib study), and 260 patients in KUH between November 2018 – May 2019 (the Single-ECG study).

The inclusion criteria were AF or sinus rhythm (SR) diagnosed by the treating physician from a 12-lead resting ECG. The exclusion criteria were a body mass index (BMI) ≥ 33 kg/m^2^ (the Afib study) or ≥ 35 kg/m^2^ (the Single-ECG study), a cardiac pacemaker, a left bundle branch block (LBBB), a right bundle branch block (RBBB), an inconclusive or a non-stable rhythm and a medical condition requiring immediate treatment. In the initial screening 182 patients were excluded; 143 due to not meeting the inclusion criteria, 21 patients declined and 18 were excluded for other reasons. After the 3-lead continuous ECG and PPG recording (see below), additional 14 patients were excluded: six due to inconclusive rhythms, two due to RBBBs and six due to inadequate PPG data. Thus, the final population consisted of 359 patients: 169 AF patients and 190 patients in SR (Figure 1).

### Data Acquisition

After the initial screening, simultaneous 3-lead ECG and PPG wrist band signals were recorded for at least 5 minutes. The 3-lead ECG was recorded with 1,000 Hz sampling frequency using a Holter ECG device (Faros 360, Bittium, Oulu, Finland) with five wet electrodes. A simultaneous PPG signal was recorded using 64 Hz sampling frequency with an Empatica E4 wrist band (Empatica Inc, Cambridge, United States). This wrist band captures optical PPG signal utilizing the blood volume pulse (BVP) method. Before the recordings, patients were resting for at least 2 min. After the rest, the ECG and the PPG signals were recorded simultaneously with the patient in the supine position.

### ECG Analysis

The final rhythm classification was based on 3-lead ECG recording interpreted by two experienced cardiologists blinded to the initial 12-lead ECG. The consensus of rhythm interpretation by the two cardiologists served as the “golden standard” for the final rhythm analysis. If no consensus was met, the patient was excluded from the study (Figure 1).

### PPG Processing and Feature Extraction

The PPG data was transferred to a MATLAB^®^ software (version R2017b) for pre-processing and analysis. The PPG data was first interpolated to 128 Hz to increase the time resolution for the beat detection. A digital zero-phase finite impulse response lowpass filter with order of 256 and a cut-off frequency of 4 Hz was used to remove high frequency noise. A PPG quality algorithm was used to identify a 1-min period of good quality PPG signal from each measurement. Only the first eligible 1-min section of each patients’ recording was utilized, and the rest of the recording was discarded. The PPG quality algorithm used acceleration measurement from the wristband to detect stable periods with no movement of the wrist and PPG amplitude variation to detect artifacts from the PPG signal. The first continuous 1-min sample of each PPG recording with at least 55 s fulfilling the above conditions was accepted for the analysis. If a good quality period was not found from the PPG recording, the patient was excluded from the final analysis (Figure 1).

**FIGURE 2:**
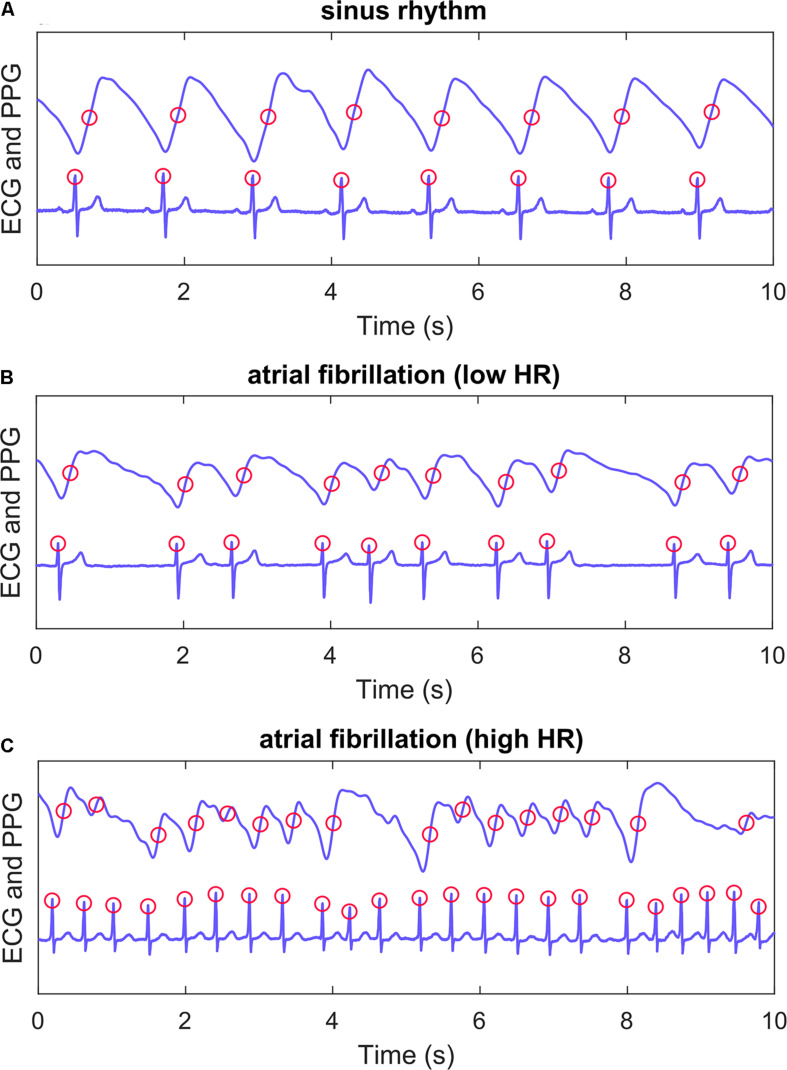
Example recordings. PPG (upper) and ECG (lower) recordings from three patients. Panel **(A)** shows a patient with sinus rhythm, panel **(B)** atrial fibrillation with lenient heart rate and panel **(C)** atrial fibrillation with high heart rate. Algorithm ECG QRS detection points and PPG pulse detection points are marked with red circles. A PIN time series was formed with detected PPG pulses for PIN-based AF detection features. ECG, electrocardiogram; PPG, photoplethysmography; HR, heart rate.

A total of ten features were calculated from the PPG signal, from which five were based on pulse interval (PIN, Figure 2) detection and four on pulse amplitude (AMP) detection. The five PIN-based variables were: mean PIN, root-mean-square values of successive differences (RMSSD), AFEvidence (AFE), Coefficient of Sample Entropy (COSEn) and turning point ratio (TPR). AFEvidence is based on relative population of segments in a 2D histogram representing dRR-intervals (Sarkar et al., 2008) and COSEn is an estimate of entropy optimized for AF detection (Lake and Moorman, 2011). Four features based on pulse amplitude were: mean AMP, RMSSD, Sample Entropy (SampEn) and TPR. These nine features are more commonly used for AF detection (Tang et al., 2017; Väliaho et al., 2019). In addition, we evaluated the performance of a more novel autocorrelation (AC) feature.

The pulses were detected from the 1-min PPG samples using a method described and validated in our previous study (Figure 2; Väliaho et al., 2019). A time series with PINs was formed from the successive pulse detections. The PIN-based features (mean, RMSSD, AFE, COSEn and TPR) were calculated from these time series.

The amplitude of each PPG pulse wave was calculated as difference of maximum and minimum amplitude in a 0.5 s window around the detected pulse. The formed amplitude time series was used to calculate the values for the AMP-based features (mean, RMSSD, SampEn and TPR). The values of TPR and AC features were used as 100-fold for better calculation accuracy (by avoiding dividing by almost 0) and estimation of odds ratios.

**FIGURE 3:**
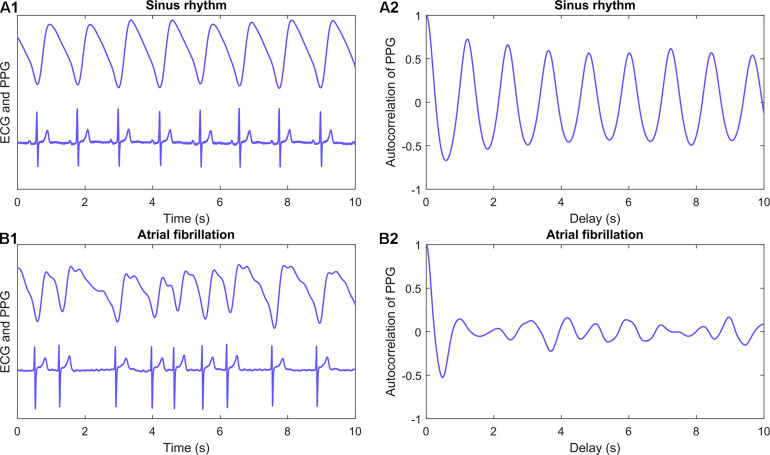
Autocorrelation. PPG (upper) and ECG (lower) recordings from a patient with sinus rhythm **(A1)** and atrial fibrillation **(B1)**. Corresponding autocorrelation values were calculated for 1-min samples of PPG signal for each patient. First 10 s of example recordings and calculated autocorrelation values **(A2** and **B2)** are shown in panels. Autocorrelation is a feature calculated straight from the signal and it requires no pulse detection. It is the correlation between a signal and its delayed copy as a function of delay. ECG, electrocardiogram; PPG, photoplethysmography.

AC is a pulse wave morphology-based feature extracted from the PPG signal. It represents the correlation between a signal and its delayed copy as a function of delay. AC describes the regularity of the PPG signal morphology without a need for detection of individual pulses from the time series (Figure 3). AC values are decreased if the shape and periodicity of the PPG pulse waves vary. The average of absolute autocorrelation values (performed over different delays) was calculated for 1-min PPG samples of each patient. The normalized value of AC for different delays can be calculated as

$$R_{l}=\frac{\left(\frac{1}{N}\,\sum_{t=1}^{N-l}x_{t}\,x_{t+l}\right)}{R_{0}}$$

where *R*_*l*_ is the value of AC for delay *l*, *R*_0_ is the value of AC with no delay, *x*_*t*_ is signal value for time index *t* and *N* is total number of samples in the signal.

### Atrial Fibrillation Detection With Univariate Predictor Models

To test how all the ten features can individually predict AF, each feature was evaluated as a univariate predictor model for detection of AF. All ten features were established in ten independent linear logistic regression models. The logistic regression uses the features to estimate the probability of the PPG sample being true positive AF. Since our dataset was balanced, the cut-off value of AF detection was set at 0.5. The performance was evaluated with 10-fold cross-validation (see below) and diagnostic performance parameter values were calculated for each univariate predictor model.

### Establishing the Multivariate Predictor Model for Atrial Fibrillation Detection

Linear logistic regression with backward feature selection procedure was used to detect independent and statistically significant features for the detection of AF with MATLAB^®^ software version R2017b. Our hypothesis was that the combined performance of some of the features is better than any of these features independently. Backward feature selection method was started with all the ten features using all the data samples and recommended significance level of 0.157 (Heinze et al., 2018). Features were removed from the model one at a time if removing them would not significantly decrease the performance of the model. A logistic regression model including only the most significant features was established. The cut-off value of AF detection was set at 0.5. The performance of this multivariate predictor model was tested with 10-fold cross-validation.

### Validation of the Predictor Models

AF data samples (*n* = 359) were randomly divided into ten sections for 10-fold cross-validation. All ten univariate predictor models and the established multivariate predictor model were validated with this method. Each time nine sections were used to train the model and one section was used to validate the prediction performance. The process was repeated ten times, with each of ten sections used only once as the validation data. The advantage of this validation method is that same samples are not used simultaneously for training and validation (reduce bias) of the predictor model, and each individual sample is used exactly once for validation. The diagnostic performance parameter values were averaged to produce a single estimation of AF prediction, including area under receiver operating characteristic curve (AUC) value, sensitivity, specificity, positive prediction value (PPV), negative prediction value (NPV) and accuracy.

The receiver operating characteristics (ROC) curve was formed for each prediction model by using the average of true and false positive rates from the 10-fold cross validation models.

### Statistical Analysis

Clinical characteristic data and PPG feature parameter values were analyzed using IBM SPSS statistics software version 25. Continuous variables between AF and SR patients were analyzed with independent-sample t-tests and categorical variables with χ^2^ tests. The significance of differences within AF and SR patients were tested with paired t-tests. All significance tests were two-tailed with *p* ≤ 0.05 considered statistically significant.

## Results

### Clinical Characteristics

The study population consisted of 359 patients (AF *n* = 169, SR *n* = 190). Patients with AF were older, had higher heart rate, more often medical history including earlier AF episodes, congestive heart failure and heart surgery, and were more often on beta-blockers, digoxin or anticoagulation therapy (Table 1). There were no adverse events related to the study recordings.

**TABLE 1:** Clinical characteristics of the patients.

	**AF group,**	**SR group,**	**Significance**	**Mean difference and**
	***n* = 169**	***n* = 190**	**(2-sided)**	**[95% CI of the difference]**
**Characteristics**				
Age, years	72.2 ± 14.3	57.9 ± 18.8	<0.001	14.29 [10.85 to 17.73]*
BMI, kg/m^2^	26.0 ± 3.9	25.8 ± 3.7	0.635	0.19 [−0.60 to 0.99]*
Sex, male	87 (51.5)	97 (51.1)	0.936	0.43 [−9.83 to 10.67]
**PPG**				
Mean heart rate, min^–1^	84.4 ± 15.0	69.8 ± 13.6	<0.001	14.59 [11.62 to 17.56]*
**Medical history**				
Atrial fibrillation	128 (75.7)	44 (23.2)	<0.001	52.58 [43.04 to 60.56]
Hypertension	112 (66.3)	96 (50.5)	0.003	15.75 [5.53 to 25.47]
Coronary artery disease	48 (28.4)	41 (21.6)	0.135	6.82 [−2.12 to 15.75]
Congestive heart failure	46 (27.2)	6 (3.2)	<0.001	24.06 [16.96 to 31.42]
Diabetes	30 (17.8)	29 (15.3)	0.525	2.49 [−5.18 to 10.31]
Cardiac surgery	22 (13.0)	9 (4.7)	0.005	8.28 [2.42 to 14.59]
Other arrhythmia	16 (9.5)	21 (11.1)	0.622	−1.59 [−7.93 to 4.93]
Structural heart disease	14 (8.3)	9 (4.7)	0.171	3.55 [−1.64 to 9.15]
**Medication**				
Anticoagulation	131 (77.5)	42 (22.1)	<0.001	55.41 [46.01 to 63.16]
Beta-blocker	125 (74.0)	74 (38.9)	<0.001	35.02 [24.99 to 43.99]
Digoxin	22 (13.0)	1 (0.5)	<0.001	12.49 [7.60 to 18.41]
Anti-arrhythmic drugs	9 (5.3)	4 (2.1)	0.103	3.22 [−0.83 to 7.88]

*Values are mean ± standard deviation and number (percentages). In the last column values are mean difference and [95% Confidence Interval of the Difference]. Values for dicotomical variables in this column (e.g., sex or hypertension) are percentages.*

AF, atrial fibrillation; CI, confidence interval; HR, heart rate; PPG, photoplethysmography and SR, sinus rhythm.

*Mean difference and [95% confidence interval of the difference] values for Age, BMI, and HR are years, kg/m^2^ and min^–1^.

### PPG Feature Comparison Between Rhythm Groups

Calculated parameter values of all ten features of the PPG signal differed between AF and SR groups (Table 2).

**TABLE 2:** Comparison of feature parameter values between atrial fibrillation and sinus rhythm groups.

**Feature**	**AF group**	**SR group**	**Significance**	**Mean difference and**
	***n* = 169**	***n* = 190**	**(2-sided)**	**[95% CI of the difference]**
	**Mean ± SD**	**Mean ± SD**		
**Pulse-interval**				
PIN_mean	0.734 ± 0.134	0.892 ± 0.166	<0.00001	−0.158 [−0.189 to −0.126]
PIN_RMSSD	0.281 ± 0.102	0.122 ± 0.111	<0.00001	0.159 [0.136 to 0.181]
PIN_AFE	58.201 ± 13.838	−26.111 ± 36.605	<0.00001	84.312 [78.432 to 90.191]
PIN_COSEn	−0.411 ± 0.554	−1.981 ± 0.511	<0.00001	1.570 [1.459 to 1.680]
PIN_TPR	61.751 ± 6.059	48.836 ± 10.984	<0.00001	12.915 [11.054 to 14.776]
**Amplitude**				
AMP_mean	64.380 ± 46.774	90.082 ± 57.042	<0.00001	−25.703 [−36.612 to −14.794]
AMP_RMSSD	27.782 ± 20.453	17.072 ± 16.800	<0.00001	10.710 [6.841 to 14.580]
AMP_SampEn	2.217 ± 1.073	1.774 ± 0.664	<0.00001	0.443 [0.260 to 0.626]
AMP_TPR	65.716 ± 6.536	57.169 ± 8.565	<0.00001	8.547 [6.951 to 10.144]
**Morphology**				
AC	4.790 ± 1.544	14.723 ± 5.306	<0.00001	−9.933 [−10.766 to −9.101]

*Values were calculated for each PPG feature for each patient individually in both rhythm groups. Numbers are mean ± standard deviation. Significance is between groups. AC, autocorrelation; AF, atrial fibrillation; AFE, AFEvidence; AMP, peak amplitude; CI, confidence interval; COSEn, coefficient of sample entropy; PIN, pulse interval; PPG, photoplethysmography; RMSSD, root mean square of successive pulse-to-pulse differences; SampEn, sample entropy; SD, standard deviation; SR, sinus rhythm and TPR, turning point ratio.*

### Univariate Predictor Models With Single Features for AF Detection

**FIGURE 4:**
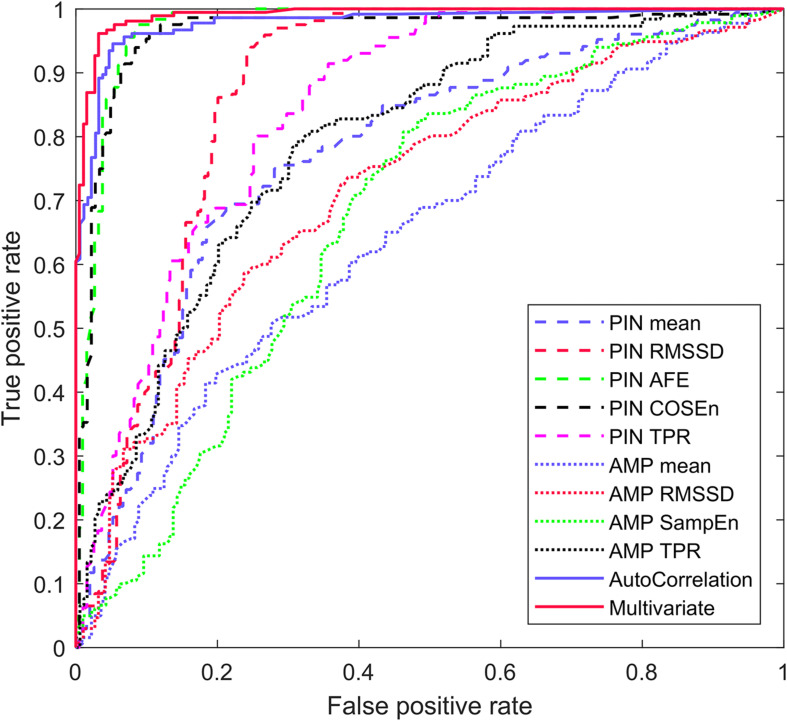
Averaged AF detection ROC curve of the univariate models and the multivariate predictor model.

The novel PPG pulse wave morphology-based AC feature detected AF as a univariate predictor model with highest AUC of 0.982 (sensitivity 95.1%, specificity 93.7%). The PIN-based AFE detected AF with AUC of 0.977 (sensitivity of 96.0%, specificity 92.9%) and PIN-based COSEn with 0.964 (sensitivity of 92.3%, specificity of 92.1%). The other seven univariate predictor models yielded lower AUC values. The averaged 10-fold cross-validated diagnostic performance parameter values for each univariate predictor models are presented in Table 3. The ROC curves for each univariate predictor models are presented in Figure 4.

**TABLE 3:** Averaged 10-fold cross-validated univariate predictor model diagnostic performance values for detection of atrial fibrillation.

**Univariate predictor models**	AUC*	**Sensitivity**	**Specificity**	**PPV**	**NPV**	**Accuracy**
**Pulse-interval**						
PIN_mean	0.780	72.2	72.2	69.6	75.0	72.1
PIN_RMSSD	0.867	77.4	80.9	78.5	79.8	78.8
PIN_AFE	0.977	96.0	92.9	93.1	97.0	94.7
PIN_COSEn	0.964	92.3	92.1	91.4	93.2	92.2
PIN_TPR	0.841	80.1	72.3	71.7	81.1	76.0
**Amplitude**						
AMP_mean	0.659	59.7	60.4	57.3	63.0	59.6
AMP_RMSSD	0.726	46.0	82.6	70.7	63.2	65.0
AMP_SampEn	0.680	48.1	72.8	61.0	61.6	61.3
AMP_TPR	0.792	72.2	73.1	71.1	74.7	72.7
**Morphology**						
AC	0.982	95.1	93.7	93.5	96.2	94.4

AC, autocorrelation; AF, atrial fibrillation; AFE, AFEvidence; AMP, peak amplitude; AUC, area under the curve; CI, confidence interval; COSEn, coefficient of sample entropy; NPV, negative predictive value; PIN, pulse interval; PPG, photoplethysmography; PPV, positive predictive value; RMSSD, root mean square of successive pulse-to-pulse differences; SampEn, sample entropy; SD, standard deviation; SR, sinus rhythm and TPR, turning point ratio. *The values are percent (%) except AUC is absolute value.

### AF Detection With the Multivariate Predictor Model

The multivariate predictor model was reduced with the backward feature selection to include only four features: PIN_AFE (*p* = 0.007), PIN_TPR (*p* = 0.008), AMP_mean (*p* = 0.031) and AC (*p* < 0.00001) (Table 4).

**TABLE 4:** Features in the multivariate predictor model.

**Feature**		**Estimate**	**SE**	**OR**	Significance (2-sided)*
Any	(Intercept)	–3.723	2.671	0.024	0.163
Pulse-interval	PIN_AFE	0.045	0.017	1.046	0.007
	PIN_TPR	0.126	0.047	1.135	0.008
Amplitude	AMP_mean	0.016	0.008	1.017	0.031
Morphology	AC	–0.771	0.171	0.463	<0.00001

*All features included in the established multivariate predictor model were statistically significant with *p* < 0.05. Intercept is the constant of the linear logistic regression model.*

AC, autocorrelation; AFE, AFEvidence; AMP, peak amplitude; OR, odds ratio; PIN, pulse interval; SE, standard error and TPR, turning point ratio.

*For logistic regression model including all four features.

The multivariate predictor model detected AF with AUC of 0.993, sensitivity of 96.4% and specificity of 96.3%. The diagnostic performance parameters of the validated multivariate predictor model are presented in Table 5. The ROC curve for the multivariate prediction model is presented in Figure 4.

**TABLE 5:** Multivariate predictor model 10-fold cross-validation diagnostic performance results in detection of atrial fibrillation.

	**Mean**	**Min**	**Max**
**Multivariate**			
AUC	0.993	0.987	1.000
Sensitivity	96.4	88.9	100.0
Specificity	96.3	90.0	100.0
PPV	96.1	88.9	100.0
NPV	96.9	88.9	100.0
Accuracy	96.4	91.7	100.0

*The multivariate prediction model consisted of PIN_AFE, PIN_TPR, AMP_mean and AC features. Mean values are averaged from ten validations with subsections. The values are percent (%) except AUC are absolute values (number).*

*AFE, AFEvidence; AMP, peak amplitude; AUC, area under the curve; Max, maximum; Min, minimum; NPV, negative predictive value; PPV, positive predictive value; PIN, pulse interval and TPR, turning point ratio.*

## Discussion

We demonstrated that the novel AC as a univariate predictor model detected AF with high sensitivity (95.1%) and specificity (93.7%) from the PPG wrist band signal. AC had the highest AUC (0.982) of all ten univariate predictor models, each containing only one PPG feature. The advantage of AC is that it requires no individual pulse detection from the PPG signal unlike all other nine features evaluated. To the best of our knowledge, this is the first time AC was assessed and validated as a predictor of AF with a PPG wrist band. The AC feature was also included in the multivariate predictor model with backward feature selection method and it turned out to be the most significant individual feature in the model (*p* < 0.00001).

In addition, our study shows that combining pulse wave morphology-based AC with PIN and AMP-based features improves the diagnostic performance of PPG wrist bands. The multivariate predictor model developed and validated in our study consisting of four PPG features detected AF with higher AUC, sensitivity and specificity (0.993, 96.4%, 96.3%) than any of the ten evaluated features as univariate predictor models independently.

Short-term AC has been used earlier for instantaneous heart rate (IHR) and R-peak detection from the ECG signal due to its noise-tolerant performance (Fujii et al., 2013). In addition, the advantage of short-term AC for wearable ECG monitoring systems is that it has low digital processing capacity requirements (Fujii et al., 2013). From the PPG signal, short-term AC has been used to estimate pulse-to-pulse interval with short 4-s time windows because it has more instability tolerance (Kashiwa et al., 2019). PPG pulse waves frequently have low peaks or varying amplitude in AF patients. This is due to loss of atrial-ventricular synchrony, impaired ventricular diastolic filling, and irregular ventricular rate. As a result, the PPG pulse detection sensitivity in patients with AF is lower compared to patients with SR, and even lower if the AF has lasted for less than 48 h (Väliaho et al., 2019). Autocorrelation as a robust and computationally very effective method can detect the absence of this morphology regularity. An obvious advantage of AC is that it recognizes AF without pulse detection.

Yan et al. used a smartphone camera to measure the PPG from the fingertip and contact-free from the face (Yan et al., 2018). They used a smartphone application utilizing a support vector machine (SVM) with the AC to detect AF from the PPG signal (Yan et al., 2018). The SVM is a machine learning technique (Kwon et al., 2019). The sensitivity and specificity were 94.7 and 95.8% for facial PPG and 94.7 and 93.0% for the fingertip PPG (Yan et al., 2018). Recently Kwon et al. reported that the SVM with the AC to detected AF with sensitivity of 93.26% and specificity of 89.60% with a pulse oximeter from the fingertip (Kwon et al., 2019). In our study the multivariate predictor model including the AC feature achieved higher sensitivity and specificity compared to both studies. Also, the method of measuring PPG was different in these studies as compared to the wrist band that was used here.

In our study we assessed the feasibility of a PPG wrist band, a commonly used method for sport and welfare purposes, for AF detection. A wide range of other devices such as smartwatches (Tison et al., 2018; Dörr et al., 2019; Guo et al., 2019; Perez et al., 2019), smartphone applications (Yan et al., 2018; Kwon et al., 2019), and chest strap ECGs (Hartikainen et al., 2019) have also been evaluated for AF detection. Recently, in the Huawei Heart study 187 912 participants were monitored with a PPG wrist band or a wristwatch (Guo et al., 2019). During the monitoring, 424 (0.23%) subjects received an irregular pulse notification and of those 262 were followed up with an ECG or 24-h Holter (Guo et al., 2019). AF was ECG-confirmed in 227 (87%) cases with the PPV 91.6% for the PPG-based algorithm (Guo et al., 2019). Correspondingly, in the Apple Heart Study PPG was recorded with a smartwatch from 419 093 participants (Perez et al., 2019). 2161 (0.52%) of subjects received PPG-based irregular pulse notifications and of those 450 were monitored with ECG patches for an average of 6.3 days (Perez et al., 2019). AF was found in 153 (34%) of the subjects (Perez et al., 2019). Only 86 individuals had irregular PPG pulse notifications during simultaneous use of an ECG patch, and AF was confirmed in 72 of these cases resulting in a PPV of 84% with the PPG smartwatch (Perez et al., 2019). Because of the study designs in both Huawei Heart Study and Apple Heart Study, sensitivity could not be assessed and thus compared to the results of our study. Perez et al. state that their PPG-based irregular pulse detection algorithm was designed to minimize false positive findings of AF and should not be used for AF screening (Perez et al., 2019). However, our algorithm produced high sensitivity (96.4%), specificity (96.3%) and PPV (96.1%) indicating PPG wrist bands could enable reliable detection of AF.

Kashiwa et al. developed a wrist band pulse wave monitor for long-term PPG monitoring that detects AF with PPG pulse frequency-based analysis (Kashiwa et al., 2019). Their AF detection was based on two statistical values: the coefficient of variation (CV) of PP values and Kolmogorov-Smirnov (KS) difference (Kashiwa et al., 2019). They detected AF with a patient average sensitivity of 81.0%, specificity of 96.4% and PPV of 86.6% with AF episodes lasting over 6 min (Kashiwa et al., 2019). Compared to Kashiwa et al., in our study, using the multivariate predictor model, the sensitivity and the PPV were higher (96.4 and 96.1%) and the specificity was equal (96.3%). Fan et al. used a novel algorithm utilizing combined PPG morphology and pulse frequency analysis to detect AF with a PPG wrist band (Fan et al., 2019). The quality of the PPG signal was assessed with a mobile phone application, and in case of rejected recording the measurement was retaken (Fan et al., 2019). In line with us, they analyzed also 1-min samples, but they extracted three samples from each patient, yielding a sensitivity of 95.36%, a specificity of 99.70% and a PPV of 99.63% for AF detection (Fan et al., 2019). The sensitivity of the multivariate predictor model in our study was slightly better but the specificity and the PPV were lower.

Recently, Tison et al. reported that PPG smartwatch was able to detect AF utilizing a deep neural network with sensitivity of 98.0%, specificity of 90.2% and PPV of 90.9% (Tison et al., 2018). They trained their method in 6,682 patients and validated it in 51 patients (Tison et al., 2018). The algorithm-based multivariate predictor model developed in our study achieved significantly higher specificity (96.3%) and PPV (96.1%) with only slightly lower sensitivity (96.4%). Also, Dörr et al. showed that an AF detection algorithm detected AF with a PPG smartwatch with a sensitivity of 93.7%, a specificity of 98.2% and a PPV of 97.8% calculated from high quality samples (Dörr et al., 2019). As compared to the other studies Dörr et al. reported high number of non-interpretable samples, more than 20% of their 1-min PPG samples remained without rhythm interpretation (Dörr et al., 2019). Our method yielded higher sensitivity and slightly lower specificity and PPV, however, by using our method there were only six (1.6%) samples which rhythm could not be interpreted.

### Limitations

AF detection was performed from 1-min PPG samples of good quality data. These samples were selected automatically by the quality algorithm. The PPG signal is susceptible to disturbances caused by movement of the optical sensor against the skin, blood pressure changes and vascular elasticity fluctuations. In our study, the PPG signal was recorded only for 5 min and from stationary patients. For detection of paroxysmal atrial fibrillation, the technology should allow longer rhythm monitoring in ambulatory patients. The quality of the data can be improved by using PPG wrist bands equipped with acceleration sensors programmed to accept only PPG signal for AF analysis when the patient is at rest and the hand is stable position. Further clinical studies are needed to assess the utility of PPG wristband in the detection of AF in long-term monitoring of ambulatory patients. The capability of the AF detection algorithms should be evaluated in a setting where the PPG signal is exposed to the artifacts caused by e.g., motion, thus describing the actual practical capability of the AF detection method in patients’ daily situations.

The effect of premature atrial (PAC) and ventricular contractions (PVC) on the AF detection was not examined in this study. The presence of premature contractions could affect the AF detection and probably impair AF detection specificity as they are probable to cause irregularity in both ECG and PPG signals. Irregular pulse during sinus rhythm with premature contractions could be falsely detected as atrial fibrillation by automated algorithms based on pulse irregularity or altering the PPG morphology.

## Conclusion

We demonstrated that the novel AC feature based on pulse wave morphology detects AF independently with high sensitivity and specificity without the need of pulse detection. In addition, we proved that combining pulse wave morphology-based features such as AC with information from pulse-interval variability it is possible to detect AF with high accuracy by using a commercial PPG wrist band.

Results indicate that PPG wrist bands accompanied with AF detection algorithm could provide an easy-access and a reliable wearable monitoring method in search of paroxysmal or asymptomatic AF.

## Data Availability Statement

The raw data supporting the conclusions of this article will be made available by the authors, without undue reservation.

## Ethics Statement

The studies involving human participants were reviewed and approved by The Ethics Committee of Kuopio University Hospital (approval number: 237/2017, 850/2018). The patients/participants provided their written informed consent to participate in this study.

## Author Contributions

E-SV, PK, JL, JH, HJ, TR, JH, MT, OS, and TM contributed to the design of the study. E-SV, TR, MC, OS, and TM contributed to the collection of the data. E-SV, PK, JL, JH, IK, HP-M, and OS performed the data analysis. E-SV and PK performed the statistical analysis. E-SV drafted the manuscript. All authors have contributed to the manuscript and approved the final version.

## Conflict of Interest

JL, TR, TM, HJ, JH, and MT are shareholders of Heart2Save company that designs ECG-based software for medical equipment. JL, MT, and HJ have a patent pending. The remaining authors declare that the research was conducted in the absence of any commercial or financial relationships that could be construed as a potential conflict of interest.

## Footnotes

1 https://clinicaltrials.gov/ct2/show/NCT03721601

2 https://clinicaltrials.gov/ct2/show/NCT03753139

## Supplementary Material

Supplementary Material 1 STARD 2015 Checklist and information about STARD 2015.
